# The Onset of Empty Nest Increases Subjective Well‐Being Amongst Middle‐Aged and Older Adults: Longitudinal National Evidence From Thailand, 2015–2022

**DOI:** 10.1111/psyg.70147

**Published:** 2026-02-18

**Authors:** Supa Pengpid, Karl Peltzer, Pratana Satitvipawee, Wasin Kaewchankha, Passakorn Suanrueang, André Hajek

**Affiliations:** ^1^ Department of Health Education and Behavioral Sciences Faculty of Public Health, Mahidol University Bangkok Thailand; ^2^ Department of Public Health Sefako Makgatho Health Sciences University Pretoria South Africa; ^3^ Department of Healthcare Administration College of Medical and Health Science, Asia University Taichung Taiwan; ^4^ Department of Medical Research China Medical University Hospital, China Medical University Taichung Taiwan; ^5^ Department of Psychology University of the Free State Bloemfontein South Africa; ^6^ Department of Psychology College of Medical and Health Science, Asia University Taichung Taiwan; ^7^ Department of Biostatistics Faculty of Public Health, Mahidol University Bangkok Thailand; ^8^ Intelligence and Information Center, National Institute of Development Administration Bangkok Thailand; ^9^ Department of Health Economics and Health Services Research University Medical Center Hamburg‐Eppendorf, Hamburg Center for Health Economics Hamburg Germany

**Keywords:** empty nest, life satisfaction, longitudinal study, negative affect, positive affect, Thailand

## Abstract

**Objectives:**

The aim of this study was to assess the longitudinal association between the transition to an empty nest and subjective well‐being (positive affect, negative affect and life satisfaction) amongst ageing adults in Thailand from 2015 to 2022.

**Methods:**

The Health, Aging and Retirement in Thailand study's four waves of longitudinal data were used. The pooled analytic sample of individuals with live children consisted of 6535 observations of men and 8521 observations of women from four study evaluations conducted in 2015, 2017, 2020 and 2022. The average age of the entire analytical sample was 68.7 years (SD = 11.9 years, range 45–107 years). Empty nest, positive affect, negative affect and life satisfaction were measured using established methods. The longitudinal association between the shift to an empty nest and subjective well‐being was estimated using linear fixed‐effects (FE) regressions.

**Results:**

Adjusted FE regressions showed a positive association between the transitions into an empty nest and positive affect in men (*β* = 0.32, *p* < 0.001) and in women (*β* = 0.17, *p* < 0.05), and a positive association between the transitions into an empty nest and greater life satisfaction in women (*β* = 0.12, *p* < 0.05) but not in men. Transitions into an empty nest were not significantly associated with negative affect in both sexes.

**Conclusions:**

The study adds to the body of knowledge on the empty nest sequelae of subjective well‐being using longitudinal data. Panel regression models are needed in future longitudinal investigations in other Southeast Asian countries to confirm our results.

## 1 Introduction

In Thailand, rapid population change brought on by low birth rates, high longevity, high rural‐to‐urban migration and shifting socioeconomic well‐being and lifestyles is causing Thai families to become more diverse in terms of both composition and function [1]. In Thailand, extended families are becoming more prevalent than nuclear families, which are currently the most common family structure. Approximately half of the population lives in extended families (such as three‐generation family = grandparents + working age children + grandchildren and skipped‐generation family = grandparents and grandchildren) while the classic nuclear family type with parents and children has declined by about half. Over the last three decades (1987–2013), the number of one‐person households has doubled, although the number of households with non‐related persons has remained relatively stable [1]. According to Thailand's 2017 Survey of Older those, 10.8% of those 60 years of age and older lived alone and family sizes are declining in tandem with the nation's notable increase in life expectancy [2, 3]. In a 1986 national survey of older adults (60 years and older) in Thailand, 77% were living with one or more children, 20% had children who were not living with their parents and 4% were childless [4]. This evolving family structure has also led to an increase of the empty nest, defined as ‘a family life course transition and postparental phase that occurs when children have moved out and left the parental home’, [5] in Thailand, Asian societies and globally [6].

Older adults with empty nests may be confronted with many obstacles in their lives that could endanger their emotional well‐being. When young people leave, the nest (family) becomes empty, leaving only the old. This type of family is known as an empty‐nest family, and the elderly members are known as empty‐nest elderly. ‘Lowered interaction, rare visits and less intensity of communication with the young adults, family conflict, strained parental relationship, abuse and neglect, poor physical health, financial stress, lack of social capital, social support and poor social network cause the higher level of mental health conditions such as loneliness, depression, stress, anxiety, fear, lower cognitive ability and lower life satisfaction’ [7]. While other studies [6] may indicate that empty nest is associated with enhanced well‐being or reduced ill health or null effects. A recent scoping review of 18 studies (all conducted in Asia and 17 studies were cross‐sectional) amongst older adults found that in the majority older empty nesters were more likely than their counterparts who were not empty nesters to experience poor mental health [8], and a systematic review of 29 cross‐sectional studies amongst older adults in China found that compared to non‐empty nests, the physiological, psychological, social and overall quality of life scores of empty nests were lower [9]. Overall, theory portrays empty‐nest status as a multifaceted life transition: risks stem from role and attachment loss, while benefits arise from strain relief and resource gains, with cultural norms, gender/cohort, intergenerational relationships and broader social–ecological factors determining whether emotional well‐being and life satisfaction decline, remain stable or improve [6, 10]. Role loss and attachment theories explain the risk of emotional distress, especially in cultures or individuals where parenting is central to identity [11, 12, 13, 14]. However, role strain relief and resource gain models highlight the potential for increased well‐being, particularly when parents have strong marital relationships, social support and opportunities for personal growth [15, 16, 17]. Social–ecological and family systems perspectives underscore the importance of broader contextual factors, such as socioeconomic status, community resources and the quality of intergenerational relationships [18, 19, 20]. Cultural and gender differences further moderate these effects, with some groups more vulnerable to negative outcomes [16, 21, 22].

Despite extensive research, gaps remain in understanding the long‐term trajectories of well‐being post‐empty nest, and cross‐cultural differences in non‐Western and non‐China contexts. Very few longitudinal studies regarding the associations between empty nest and subjective well‐being have been carried out, including in Thailand, which led to this study. An understanding of the well‐being consequences of empty nest on subjective well‐being amongst ageing adults may aid in the development of social and health policies for the management of empty nest consequences amongst older persons. In the few longitudinal studies investigating empty nest and subjective well‐being found mixed results. For instance, in Australia, the majority of mothers reported that their moods improve and their daily difficulties decrease when the final child leaves the home [23]. After controlling for baseline sociodemographic and health measures, having all of your children out‐migrated at baseline predicted lower odds of depression in a demographic site in rural Thailand compared to having none or some of your children out‐migrated [24]. The shift to an empty nest was no longer linked to a change in loneliness or depressive symptom scores in Germany, according to the adjusted model [22]. In China, elderly adults who become empty nesters may not have any short‐term effects on their mental health. However, over time, their mental health would suffer if they did not have regular contact with their children [25]. Another study amongst older adults in China found that empty nesters are more likely to experience depression and have higher levels of it than those who are not. At older ages, the growth rate of depression amongst older adults who are empty nesters is larger than that of elderly people who are not [26]. In a longitudinal study in China (2008 and 2011) amongst older adults showed that an empty nest was significantly negatively associated with psychological health, including positive affect [27].

Comparing these results from different countries (middle‐income, China and Thailand, and high‐income, Australia and Germany) and cultural (collectivism in China and rural Thailand, and individualism in Germany and Australia) contexts, mixed results were found on the relationship between empty nest and subjective well‐being. Therefore, we hypothesise mixed results of the effects of empty‐nest status on subjective well‐being amongst people living in Thailand. The aim of this study was to assess the longitudinal association between the transition to an empty nest and subjective well‐being (positive affect, negative affect and life satisfaction) amongst ageing adults in Thailand from 2015 to 2022.

## 2 Methods

### 2.1 Participants

Four waves of Health, Ageing and Retirement in Thailand (HART) studies conducted in Thailand in 2015, 2017, 2020 and 2022 were subjected to longitudinal analysis. Using a multi‐stage national sample approach, one adult (over 45) was chosen at random from each household (as the inclusion criterion) and interviewed in the home; further details are given elsewhere [28]. The pooled analysis of people with live children consisted of 13 560 observations, 6077 observations of men and 7483 observations of women from four study assessments conducted in 2015, 2017, 2020 and 2022. Of the 5031 participants at baseline, 455 had passed away, 40 had declined and 2196 could not be reached during the four waves from 2015 to 2022, yielding a response rate of 56%. Participants provided signed informed consent, and the study's protocol was approved by the National Institute for Development Administration's Human Research Ethics Committee (ECNIDA 2020/00012).

### 2.2 Measures

#### 2.2.1 Outcome Variables

Positive affect (0–6) and negative affect (0–24) were assessed with two items and eight items, respectively, of the Center for Epidemiologic Studies Depression Scale (CES‐D‐10) [29]. Higher scores indicated higher positive or negative affect; Cronbach's *α* was 0.82 and 0.79 for positive affect and negative affect, respectively. The construct validity of the positive and negative affect factors of the CES‐D‐10 is supported by factor analyses, which generally show that these are two distinct, relatively independent constructs [29, 30, 31]. A depression/well‐being continuum is measured by the CES‐D [32].

The question, ‘Overall, how satisfied are you with your quality of life?’ was used to gauge life satisfaction using a visual analog scale, expressed on a scale of ‘0 (*very poor*) to 10 (*excellent*)’. Measures of life satisfaction with just one question are just as valid and dependable as those with multiple items [33, 34].

#### 2.2.2 Independent Variables

At each wave from those participants who said they had living children, they were asked about child 1, 2, 3, and so forth, ‘Does the child live with the main respondent?’ (yes/no). Those who answered ‘no’ to Child 1 to Child 14 were defined as empty nest, as in previous studies [22]. Furthermore, participants whose children were not living with them were asked about the ‘Location of the child's home and the parent's home’, with the response options (1) ‘Houses next to each other/near each other’, (2) ‘Same village/in the same municipality’, (3) ‘Different districts but same province’, (4) ‘Different provinces’, (5) ‘Live abroad’ and (6) ‘Unknown’.

Sociodemographic factors included residency (rural/urban), age in years (45–107 years), sex (female/male), number of live children, marital status, educational attainment and subjective economic and work status. ‘Are you currently working for income?’ was the inquiry used to gauge work status (yes/no). The former was evaluated by asking, ‘How satisfied are you with your economic situation?’ greater scores between 0 and 10 indicate a greater economic status.

Money received from offspring in the past year was assessed with two questions: (1) Average amount received regularly from child (Baht/year) and (2) Amount received on special occasions from child (Baht/year). Amounts were coded as 1 = 0 Baht/year, 2 = < 12 000 Baht/year and 3 = ≥ 12 000 Baht/year (on average in 2018 = 32.3 Thai Baht to 1 US dollar).

Four questions were used to gather information about religious involvement. For example, ‘Prayer in the morning/before going to bed’. Higher scores indicated stronger religious commitment; Cronbach's *α* was 0.83. The response options ranged from 0 = never to 3 = always, for a total range of 0–12.

Social participation was defined as engaging in at least one of the following six activities: sports, music, art and culture, social, volunteer and/or political activities (yes/no). Its Cronbach's *α* value is 0.68.

Self‐rated physical health status was assessed with the question, ‘In general, how would you rate your physical health status?’ reported on a ‘0 (*very poor*) to 10 (*excellent*)’ scale.

A five‐item Activities of Daily Living (ADL) scale (dressing, washing, eating, bathing or showering and other activities) was used to measure functional constraints of ADLs. Responses ranged from (0) ‘Can do every step myself without needing help’, (1) ‘Need help sometimes with some steps’, (2) ‘Need help with some steps’, (3) ‘Need help sometimes with every step’ and (4) ‘Need help every time with every step’. Higher ratings indicated more functional restrictions. The total scores ranged from 0 to 20. The Cronbach's *α* was 0.93.

### 2.3 Statistical Analysis

Descriptive statistics were used to describe the sample. The longitudinal relationships between the shift to an empty nest and subjective well‐being (positive affect, negative affect and life satisfaction) in men and women were estimated for the four study waves from 2015 to 2022 using linear fixed‐effects (FE) regressions. The choice of FE regression was confirmed by the Hausman specification test between the FE and random‐effects models (for the three dependent variables, sex stratified: *p* < 0.001). This model is especially good at eliminating biases caused by systematic links between explanatory variables and time‐constant factors (both observed and unobserved), such as genetics, because individual FE account for all person‐level variability [35]. Thus, the problem of unobserved heterogeneity is much reduced when FE regressions are used. Based on previous work, independent variables were incorporated [22, 23, 24, 25, 27].

Only complete cases (missing cases were less than 3% in study variables) were analysed, and *p* < 0.05 was considered significant. We computed cluster‐robust standard errors [36]. There were no multicollinearity problems in this analysis, as indicated by the VIF scores (≤ 1.56). StataSE 18.0 (College Station, TX, USA) was used for statistical analysis.

## 3 Results

### 3.1 Sample Characteristics

The pooled sample characteristics for participants included in FE regression analysis (stratified by sex) are shown in Table 1. Amongst the total analytic sample including those with living children, the average age was 68.7 years (SD = 11.9 years, ranging from 45 to 107 years). Stratified by sex, average positive affect was 3.5 (SD = 2.1) in men and 3.7 (SD = 2.1) in women; the mean negative affect was 2.2 (SD = 2.8) in men and 2.6 (SD = 3.1) in women; and the average life satisfaction was 7.97 (SD = 1.5) in men and 7.89 (SD = 1.5) in women. A total of 1029 individuals underwent the transition to an empty nest during the course of the four study waves from 2015 to 2022.

**TABLE 1:** Sample characteristics for individuals (13 560 observations) and stratified by sex (men: *n* = 6077 observations; women: *n* = 7483 observations) (Waves 1–4, pooled).

Variables	Sample	Men	Women
Age in years: mean (SD)	68.7 (11.9)	68.8 (11.7)	68.7 (12.1)
Marital status			
Married/cohabiting	8266 (61.4)	4723 (78.2)	3543 (47.7)
Single/divorced/separated/widowed	5196 (38.6)	1318 (21.8)	3878 (52.3)
Working	5111 (38.6)	2686 (45.7)	2425 (33.0)
Subjective economic status (0–10)	6.58 (1.9)	6.63 (1.9)	6.53 (1.9)
Urban residence (rural residence)	6807 (50.2)	3046 (50.1)	3761 (50.3)
Education (> primary)	2329 (17.2)	1359 (22.4)	970 (13.0)
Social engagement	5218 (38.5)	2336 (38.4)	2882 (38.5)
Religious involvement (0–12)	6.29 (3.5)	5.91 (3.5)	6.61 (3.4)
Better self‐rated physical health (0–10)	7.21 (1.8)	7.26 (1.8)	7.17 (1.8)
Functional limitations (0–20)	0.32 (1.7)	0.29 (1.6)	0.35 (1.8)
Money received from children			
0	5990 (44.2)	2804 (46.2)	3186 (42.6)
< 12 000 Baht/year	3217 (23.8)	1439 (23.7)	1778 (23.8)
≥ 12 000 Baht/year	4338 (32.0)	1830 (30.1)	2508 (33.6)
Empty nest^a^	10 116 (74.6)	4452 (73.3)	5664 (75.7)
Positive affect (0–6)	3.58 (2.1)	3.47 (2.1)	3.66 (2.1)
Negative affect (0–24)	2.43 (3.0)	2.22 (2.8)	2.60 (3.1)
Life satisfaction (0–10)	7.93 (1.5)	7.97 (1.5)	7.89 (1.5)

^a^ Majority of children lived within the same province, followed by another province and less than 3% lived abroad.

### 3.2 Regression Analysis

Linear FE regressions adjusted for potential confounders showed a positive association between the transitions into an empty nest and positive affect in men (*β* = 0.32, *p* < 0.001) and in women (*β* = 0.17, *p* < 0.05), and a positive association between the transitions into an empty nest and positive life satisfaction in women (*β* = 0.12, *p* < 0.05) but not in men. Transitions into an empty nest were not significantly associated with negative affect in both sexes (see Table 2).

**TABLE 2:** Longitudinal association between onset of empty nest and subjective well‐being, overall and by sex.

Independent variables	Positive affect—men	Positive affect—women	Negative affect—men	Negative affect—women	Life satisfaction—men	Life satisfaction—women
Unadjusted						
Onset of empty nest (Ref: Living with children)	0.44 (0.1)***	0.24 (0.09)**	−0.10 (0.13)	0.04 (0.13)	−0.07 (0.07)	0.13 (0.06)*
Adjusted^a^						
Onset of empty nest (Ref: Living with children)	0.32 (0.10)***	0.17 (0.09)*	−0.11 (0.13)	0.03 (0.13)	−0.07 (0.06)	0.12 (0.06)*
Potential confounders	✓	✓	✓	✓	✓	✓
Constant	3.52 (0.28)***	3.53 (0.22)***	4.52 (0.43)	4.48 (0.30)***	5.04 (0.19)***	4.77 (0.15)***
Observations	5815	7246	5819	7252	5840	7287
Number of individuals	2548	3012	2548	3014	3275	3026
*R* ^2^	0.08	0.07	0.04	0.03	0.17	0.16

*Note:* Results of linear fixed effects regression analyses (Waves 1–4), *β* coefficients are reported; cluster‐robust standard errors are in parentheses.

^a^ Potential confounders include age, residence status, marital status, work status, subjective economic status, self‐rated physical health status, functional limitations, religious involvement, social engagement and money received from offspring.

*** *p* < 0.001.

** *p* < 0.01.

* *p* < 0.05.

In sensitive analysis, the co‐residence variable included (1) ‘living with the main respondent’ or (2) ‘Houses next to each other/near each other’ in addition to living in the same household, reducing the empty nest variable downwards, but similar regression results were produced.

## 4 Discussion

For the first time, the study quantified the relationships between moving to an empty next and subjective well‐being (positive affect, negative affect and life satisfaction) amongst middle‐aged and older persons in Thailand using nationwide, longitudinal data from 2015 to 2022. In adjusted FE regression analysis, empty nest parenthood was associated with an increase in positive affect in both sexes, and an increase in life satisfaction in women but not men, while no significant association was found between transitioning to an empty nest and negative affect, in both sexes.

Consistent with several longitudinal studies in women in Australia [23], in a demographic site in rural Thailand [24] and in a study in China [37], this study found a positive association between transitioning to an empty nest and subjective well‐being. The positive association between empty nesting and well‐being in Thailand may stem from cultural factors like strong family interdependence shifting towards greater parental autonomy, relief from intense parenting burdens (role strain relief) [38], increased time/energy for self‐care/hobbies/spousal relationships and a potential re‐engagement in social/community activities, aligning with Thai cultural values that can support older adults' new roles and personal growth [6, 39].

Further, the study showed that empty nest transition was associated with higher life satisfaction in women, which is in agreement with some studies in China [40, 41]. Women in Thailand may experience higher life satisfaction after their children leave home than men due to a combination of role strain relief and the ability to find new social engagement and identity [16, 42]. In contrast, men's identity is often more heavily tied to their traditional role as a provider, which is not directly impacted by the empty nest transition in a positive way [43].

Finally, the study found no significant association between transitioning to an empty nest and negative affect in both sexes, which is in line with a study in Thailand and in Germany [22]. The parental role loss model [38] may have a less significant effect on negative emotion in Thai culture primarily because of strong traditional values, such as filial piety and intergenerational support, which ensure that parental roles and family bonds continue and even strengthen after children leave the home [44]. In rural Thailand, for example, parents show lower levels of depression when children live further from rather than closer to the parental home [24] because their successful migration confers a sense of pride and higher social status for the parents, thus enhancing ego integrity [42] and mitigating the impact of role loss. This example highlights the cultural specificity of norms and expectations surrounding nest‐leaving [6].

### 4.1 Study Strengths and Limitations

The study incorporates known metrics from the Korean Longitudinal Study on Aging (KLOSA) and the Health and Retirement Study (HRS), as well as a longitudinal, national community‐dwelling population. Using panel FE regression models, we reduced the problem of unobserved heterogeneity. A further limitation was that only one item was used to quantify life satisfaction. Further research is required to obtain more thorough measurements. However, single‐item life satisfaction measures [19, 20] have been found reliable and valid as multi‐item measures. The fact that all data were evaluated using self‐report is one study drawback; objective measurements must to be incorporated into future evaluations as well. Although efforts have been made to control for a number of time‐varying confounders, reverse causality cannot be ruled out. Some variables, such a frequency of contact with children, were not assessed in all survey waves in this study and were therefore excluded from this analysis.

## 5 Conclusions

The study adds to the body of knowledge on the empty nest sequelae of subjective well‐being using longitudinal data. In adjusted FE regression analysis, empty nest parenthood was associated with an increase in positive affect in both sexes and an increase in life satisfaction in women but not men, while no significant association was found between transitioning to an empty nest and negative affect, in both sexes. Future longitudinal studies using panel regression models in other Southeast Asian countries are required to confirm our results.

## Funding

The Health, Aging and Retirement in Thailand (HART) study is sponsored by Thailand Science Research and Innovation (TSRI) and the National Research Council of Thailand (NRCT).

## Ethics Statement

The study received ethical approval from the ‘Ethics Committee in Human Research, National Institute of Development Administration—ECNIDA (ECNIDA 2020/00012)’ and participants provided written informed consent. All experiments were performed in accordance with relevant guidelines and regulations (such as the Declaration of Helsinki).

## Conflicts of Interest

The authors declare no conflicts of interest.

## Data Availability

Data is publicly available at Health, Aging, and Retirement in Thailand (HART): https://hart.nida.ac.th/download‐center/.
